# New Type of Spectral Nonlinear Resonance Enhances Identification of Weak Signals

**DOI:** 10.1038/s41598-019-50767-z

**Published:** 2019-10-01

**Authors:** Rongming Lin, Teng Yong Ng, Zheng Fan

**Affiliations:** School of Mechanical and Aerospace Engineering, Nanyang Technological University, Singapore, Singapore

**Keywords:** Aerospace engineering, Techniques and instrumentation

## Abstract

Some nonlinear systems possess innate capabilities of enhancing weak signal transmissions through a unique process called Stochastic Resonance (SR). However, existing SR mechanism suffers limited signal enhancement from inappropriate entraining signals. Here we propose a new and effective implementation, resulting in a new type of spectral resonance similar to SR but capable of achieving orders of magnitude higher signal enhancement than previously reported. By employing entraining frequency in the range of the weak signal, strong spectral resonances can be induced to facilitate nonlinear modulations and intermodulations, thereby strengthening the weak signal. The underlying physical mechanism governing the behavior of spectral resonances is examined, revealing the inherent advantages of the proposed spectral resonances over the existing implementation of SR. Wide range of parameters have been found for the optimal enhancement of any given weak signal and an analytical method is established to estimate these required parameters. A reliable algorithm is also developed for the identifications of weak signals using signal processing techniques. The present work can significantly improve existing SR performances and can have profound practical applications where SR is currently employed for its inherent technological advantages.

## Introduction

Stochastic Resonance (SR) is a unique phenomenon of certain nonlinear systems whereby a generally feeble input such as a weak signal is amplified and optimized by the assistance of an entraining white Gaussian noise, or other entraining signals such as sinusoids. Due to its simplicity and robustness, it is widely believed that SR has been implemented by mother nature on a broad spectrum of physical and biological systems in every feature size scale, and has attracted continuous interdisciplinary research interests over the last 3 decades from scientists, engineers, biologists and medical practitioners who nowadays routinely deploy SR as a useful tool for their specific applications. To date, numerous new emerging developments and applications have been discovered and reported as discussed in some of the key reviews on the subject matter^1–3^, since the concept of SR was first put forward as a possible explanation of the periodic recurrences of the Earth’s ice ages^4–6^. Existing research has been largely focusing on the theory and the mechanism, the experimental realizations, as well as important practical applications of SR with an objective to better understand and exploit SR for its potential technological advantages.

The first experimental demonstration of SR was accomplished in 1983 based on a noise-driven electronic circuit known as Schmitt trigger^7^ in which signal-to-noise ratio was first proposed to characterize SR performance. A few years later, notable SR effect was also observed in a bistable-ring laser experiment^8^ which ignited great enthusiasm among physicists. Subsequently, SR had been reported in a wide variety of physical and biological systems such as the motion of a Brownian particle^9^, superconducting quantum interface devices (SQUID)^10–12^, voltage dependent ion channels^13^, a single-walled carbon nanotube ion channel^14^, excitable GaAs superlattice^15^, bistable nanomechanical oscillators^16^, nanoscale resonant-tunneling diodes^17^, vertical-cavity surface-emitting lasers subject to time-delayed optical feedbacks^18^, receptors in cricket^19^ and crayfish^20^, as well as feeding behavior of paddle fish^21^. These indicate that SR has since become a truly interdisciplinary research and increasing evidences have further shown that SR may affect research in medical and environmental sciences.

Once the ubiquitous existence of SR is established through numerous experimental demonstrations, extensive research on the theory and mechanism of SR has followed. Nonlinear bistable systems with double-well potentials were the classical examples which were extensively analyzed for their SR behaviour^22–26^. It was generally believed that the manifestation of SR effect in bistable systems was attributed primarily to the inherent switching behavior between the two competing stable equilibriums. Subsequently however, it was discovered that the possession of a double-well potential was not essential for SR to occur since some nonlinear systems with single-well potentials were also found to exhibit prominent SR effect when properly tuned^27–29^. Furthermore, recent theoretical work has shown that SR can occur even in a simplest possible system which consists of nothing more than a signal and an entraining noise with a threshold-triggered device^30–34^, or with a basic integrate-and-fire dynamic mechanism typically found in the modeling of information transmission among neurons^35–37^. Theoretically, it has been firmly established that SR occurs when a feeble subthreshold signal is subjected to an additive noise, which enables threshold crossings. However, very recent theoretical analysis has shown that signal enhancement can also be achieved when a suprathreshold signal passes through a noisy summing network of threshold devices^38–41^, a phenomenon often called superathreshold stochastic resonance (SSR), even though in this case the signal to be identified is substantial in its own strength.

Today, we know SR is a general nonlinear phenomenon which occurs in a wide range of nonlinear systems. The noise-mediated detection and transmission of weak signals have been found to play significant roles in many technological and biological applications. They have been successfully applied in the designs of measurements^42^, in enhanced vibration energy harvesting^43^ and machine condition monitoring and fault diagnosis^44^. More importantly, sensory neurons are typically modeled as nonlinear threshold-controlled biological systems and the simple fact that SR exists in these systems offers, for the first time, direct and convincing explanation the intriguingly exquisite sensitivity of some animals to weak coherent signals. Massive research has been undertaken to date on applications of SR to the modeling of neural systems, as witnessed in some of the recent reviews^45,46^. However, what limits the success of these applications has been that when broad band noise is assumed as an entraining signal, the improvement in signal-to-noise ratio tends to be rather limited. Though there is legitimate reason why noise was first recommended as a choice for the entraining signal, there has been no evidence to suggest that other forms of signals cannot be used, especially if these can further enhance the SR effect. For the identification of weak signal which is the major application of SR, the entraining signal, which is a user selected external input, should always be so decided as to ensure best signal enhancement. For applications in biological systems on the other hand, it may be more realistic to assume that these systems are capable of also internally generating other forms of entraining signals such as sinusoids^47^ in order to achieve best sensibility of weak coherent signals, thereby ensuring their own adaptability and survivability. Against the backdrop of such reasoning, high frequency sinusoids were suggested as alternative entraining signals to replace random noise and as a result, very similar performance of SR has been observed^48,49^. Such similar performance can be largely expected since, to the weak signal, the fast-changing high frequency entraining sinusoid behaves essentially just like noise.

Alongside with the huge research effort in applying SR to weak signal detections and identifications, there has been considerable interest in reaping the potential benefits of chaotic behavior of nonlinear dynamic systems in the presence of weak signal inputs. Long term outputs of chaotic nonlinear dynamic systems are found to be extremely sensitive to slight perturbations in inputs and such high sensitivity can be effectively employed to detect weak signals by superimposing them to the inputs and comparing the resultant outputs. The intermittent chaotic motion of Duffing’s oscillator was examined for possible identification of weak signals^50^. A new signal detection and estimation method was developed based on the intermittency transition between order and chaos^51^. An effective algorithm was discussed and applied to extract weak signals drowned beneath the noise floor^52^. Based on chaotic oscillators, an information fusion technique was developed for weak periodic signals^53^. Further, for chaos in microsystems, a chaotic MEMS resonant beam sensor was developed for weak signal applications^54^. Further, based on convex optimization, weak harmonic signals can be detected from strong chaotic interferences^55^. To further randomize chaotic responses, a series of chaotic resonators can be connected and synchronized for weak signal applications^56^. More recently, nonisochronous Hopf oscillators were examined for their potentials for weak signal identifications^57^.

Though weak signals can be possibly detected using chaotic characteristics of nonlinear system responses, subsequent identification of the signal remains a problem since chaos itself means unpredictability and as a result, the exact identification of the very weak signal embedded within largely unpredictable chaotic sequence of data becomes practically almost impossible. In the applications of SR however, it is generally believed that it is capable of identifying weak embedded signals. Mathematically, SR can be viewed as a process of nonlinear modulations and intermodulations among the weak and the entraining signals, resulting in energies being transferred from the latter to the former to improve its sensibility, as we will discuss further in much more rigor later in the paper. Unfortunately however, when either Gaussian white noise or high frequency sinusoids are chosen as entraining signals, desired nonlinear modulations and intermodulations are somewhat discouraged due to the wide-spread of energy in the former and the large separation in frequency (from that of the signal) in the later, leading to very limited gain of signal enhancement. Here we propose a new and much more effective strategy for driving a system into strong spectral resonances and we report that orders of magnitude better signal enhancement which has been achieved by employing sinusoidal entraining signals with frequencies in the same range as that of the weak signal. By allowing the entraining frequency to be tuned in the range of the weak signal, the underlying mechanism of spectral resonances tend to facilitate greatly increased nonlinear modulations and intermodulations, and their associated energy transfers, thereby strengthening the weak signals. Such underlying physical mechanism governing the behavior of spectral resonances is examined, revealing the inherent advantages of the proposed spectral resonances over the existing implementation of SR. A wide range of parameters have been found to exist for the optimal enhancement of a given weak signal and an analytical method has been developed which can be applied to estimate these required parameters. A practically reliable algorithm has been proposed for the effective identification of weak signals using novel signal processing techniques. The work provides significantly improved SR performances, as well as effective methods for weak signal identifications, and can have profound practical applications where SR mechanisms are currently employed for its potential technological advantages.

## Results

### Significantly improved performance of the proposed spectral resonance

**Figure 1:**
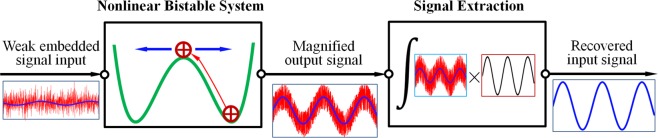
Fundamental mechanism of SR and signal extraction.

Current stochastic resonance is often considered for the simplest possible nonlinear system representing an over damped bistable oscillator described by,

1 $$\dot{x}=-\frac{d}{dx}U(x)+A\,\sin (\omega t+\alpha )+F(t)$$

where *U*(*x*), *A*sin(*ωt* + *α*) and *F*(*t*) are the potential function of the system, the weak periodic input signal to be identified and the entraining signal. The potential usually takes the form of *U*(*x*) = −(1/2)*ax*^2^ + (1/4)*bx*^4^ in which *a* = 1 and *b* = 1 are assumed to be dimensionless in this study since other values of system parameters *a* and *b* can be transformed to this standard form by making use of rescaled variables^2^. The entraining signal *F*(*t*) can assume the form of either a random noise $F(t)=\sqrt{2D}\xi (t)$ in which *ξ*(*t*) is the Gaussian white noise of standard normal distribution and *D* is its strength, or a sinusoid *F*(*t*) = *C* sin Ω*t* in which frequency *Ω* was originally proposed to be Ω >> *ω*^48^. The fundamental mechanism of SR, together with the subsequent signal extraction, can be schematically illustrated in Fig. 1 in which theapplied entraining signal is supposed to lift the system just out of its potential wells by overcoming the potential barrier so that the embedded weak signal becomes capable to assist the crossings between the two competing stable equilibriums, thereby enhancing the strength and the sensibility of the weak signal before it is identified.

**Figure 2:**
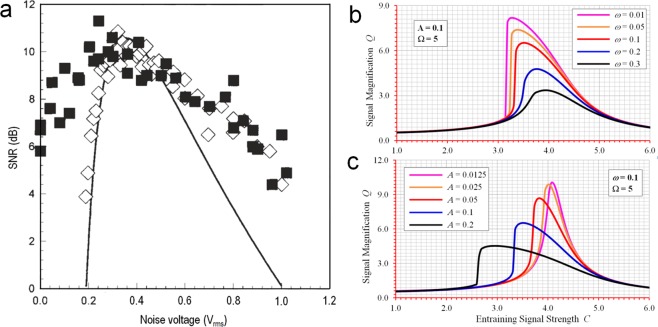
Current performances of SR with limited signal enhancement. (**a**) Signal-to-noise ratios from ref.^1^, results of experiment with crayfish mechanoreceptors (filled squares ▪) compared to the electronic Fitzhugh-Nagumo simulation (diamonds ◆) and the theoretical results (solid curve ^__^). (**b**,**c**) Signal magnification factors with entraining signal being high frequency sinusoid, numerically reproduced from ref.^48^.

Though many important practical applications have been increasingly identified, the current implementation of SR using either random or sinusoidal signal with high frequency as an entraining signal is only capable of achieving very limited gain of signal enhancement as shown in Fig. 2. An order of magnitude improvement in signal-to-noise ratios in the case of random entraining signal (Fig. 2a), or in signal magnification factors in the case of sinusoidal entraining signal with high frequency (Fig. 2b,c), is roughly what has been achieved to date for the standard bistable system of (1) when SR is provoked and such performances are certainly very inadequate for many demanding applications where very weak signals need to be identified.

**Figure 3:**
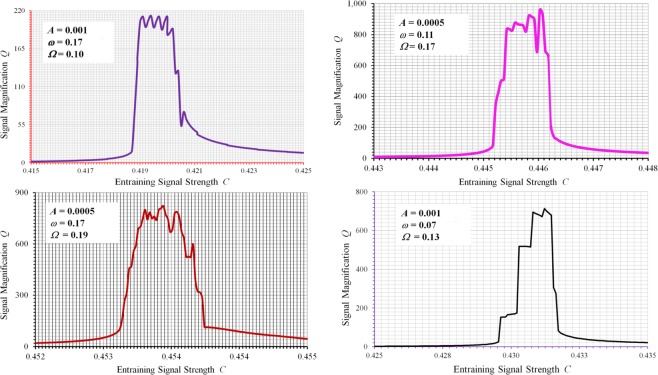
Magnification factors showing strong spectral resonances at different weak input and entraining signal parameters. (**a**) *A* = 0.001, *ω* = 0.17 and *Ω* = 0.10. (**b**) *A* = 0.0005, *ω* = 0.11 and *Ω* = 0.17. (**c**) *A* = 0.0005, *ω* = 0.17 and *Ω* = 0.19. (**d**) *A* = 0.001, *ω* = 0.07 and *Ω* = 0.13 (all parameters are non-dimensionalized).

The choice of random or sinusoidal signal with high frequency as an entraining signal has been proven to be unfortunate. Mathematically, SR is essentially a manifestation of nonlinear modulations and intermodulations among the weak and the entraining signals, leading to exchanges in energy and hence improvement in the strength of the weak signal. However, such exchanges of energy are not best encouraged when a random signal or a sinusoidal signal with high frequency is employed, since in both cases very little energy is available for exchange around the frequency band of the weak signal, resulting in very limited gain of signal enhancement. To further facilitate energy exchanges and hence to further enhance SR performance, it becomes clear that sinusoidal entraining signals with frequencies in the same range as that of the weak signal need to be employed. By allowing the frequency of the entraining signal to be tuned in the same range as that of the weak signal, the underlying mechanism of SR has undergone significant change so as to facilitate maximum possible nonlinear modulations and intermodulations and hence the subsequent energy transfers. It becomes now truly possible to tune a stochastic resonator to its true intrinsic resonance of much stronger strength which we call spectral resonance due to the fact that the entraining signal in this case is deterministic not random. What we have observed and benefited from the existing SR studies to date is in fact just the tip of the iceberg as far as the true potentials of spectral resonances are concerned. Significant improvement over performance becomes readily obtainable once the system is tuned at/near its strong spectral resonances. For every weak signal to be identified, there exists a wide range of parameters in which we can tune the entraining signal so that much stronger spectral resonance can be induced. Once the system is driven into its spectral resonance, the signal magnification factor *Q*, which is defined as the ratio between the magnitudes of the output and the input at signal frequency *ω*, can become very high, as shown in Fig. 3 for various parameters of the weak signal and the entraining signal. The initial condition required in the computation of system responses for all case studies is set as *x*(0) = 0 when *t* = 0. As compared with what have been achieved to date as shown in Fig. 2, orders of magnitude further improvement over signal enhancement becomes possible. The results also demonstrate that although we have benefitted considerably from stochastic resonances, existing studies have not yet addressed the huge hidden potential of strong spectral resonances.

**Figure 4:**
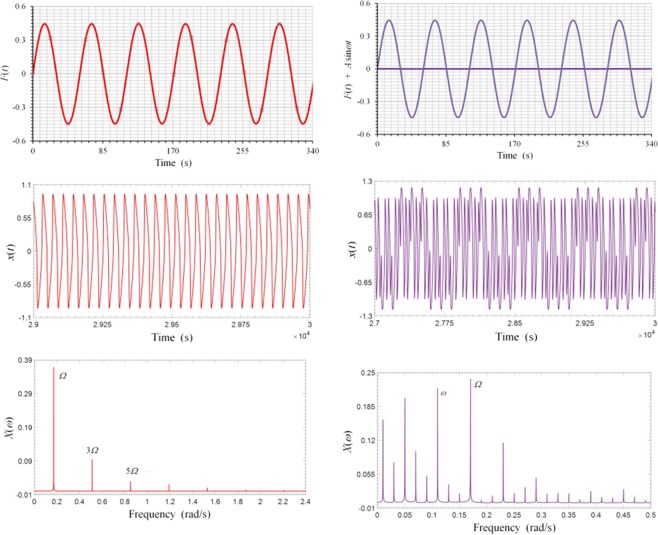
Periodic response under sinusoidal entraining input and strong spectral resonance under combined signal and entraining inputs. (**a**) Entraining signal. (**b**) Response signal. (**c**) Spectrum of response signal in (**b**). (**d**) Combined signal and entrain inputs. (**e**) Response of stochastic resonance. (**f**) Spectrum of response signal in (**e**).

To see what has actually happened at the signal level when strong spectral resonance occurs, we consider a specific case in which the weak signal has an amplitude *A* = 0.0005 and a frequency *ω* = 0.11, and the entraining signal an amplitude *C* = 0.44605 and a frequency Ω = 0.17. When the weak signal is absent, the waveform of the system response appears to be very regular, with a dominant fundamental frequency which is the driving frequency Ω = 0.17, together with odd harmonic components, which are normally expected from such a highly nonlinear system, as shown in Fig. 4a–c. When the weak signal is added however, even though its magnitude is only about 0.1% of that of the entraining signal, much of the entraining energy has been transferred to the weak signal through nonlinear modulations and intermodulations greatly boosted at spectral resonance. As we can see from Fig. 4d–f that the waveform of the response in this case becomes much more complex, with its spectrum showing not only the two input frequencies and their harmonics, but many lower sub-harmonic frequency components with frequency values much lower than either of the input frequencies. The emergence of substantial sub-harmonic components in overall system response is the hallmark of strong spectral resonance and it can be used as a criterion in practice to ascertain whether a true stochastic resonance has occurred. In addition, it is worth noting that the response frequency component at the frequency of the weak signal has grown to become very substantial despite its feeble origin, as shown in Fig. 4f, indicating that the weak signal has been greatly magnified in the process of strong spectral resonance.

**Figure 5:**
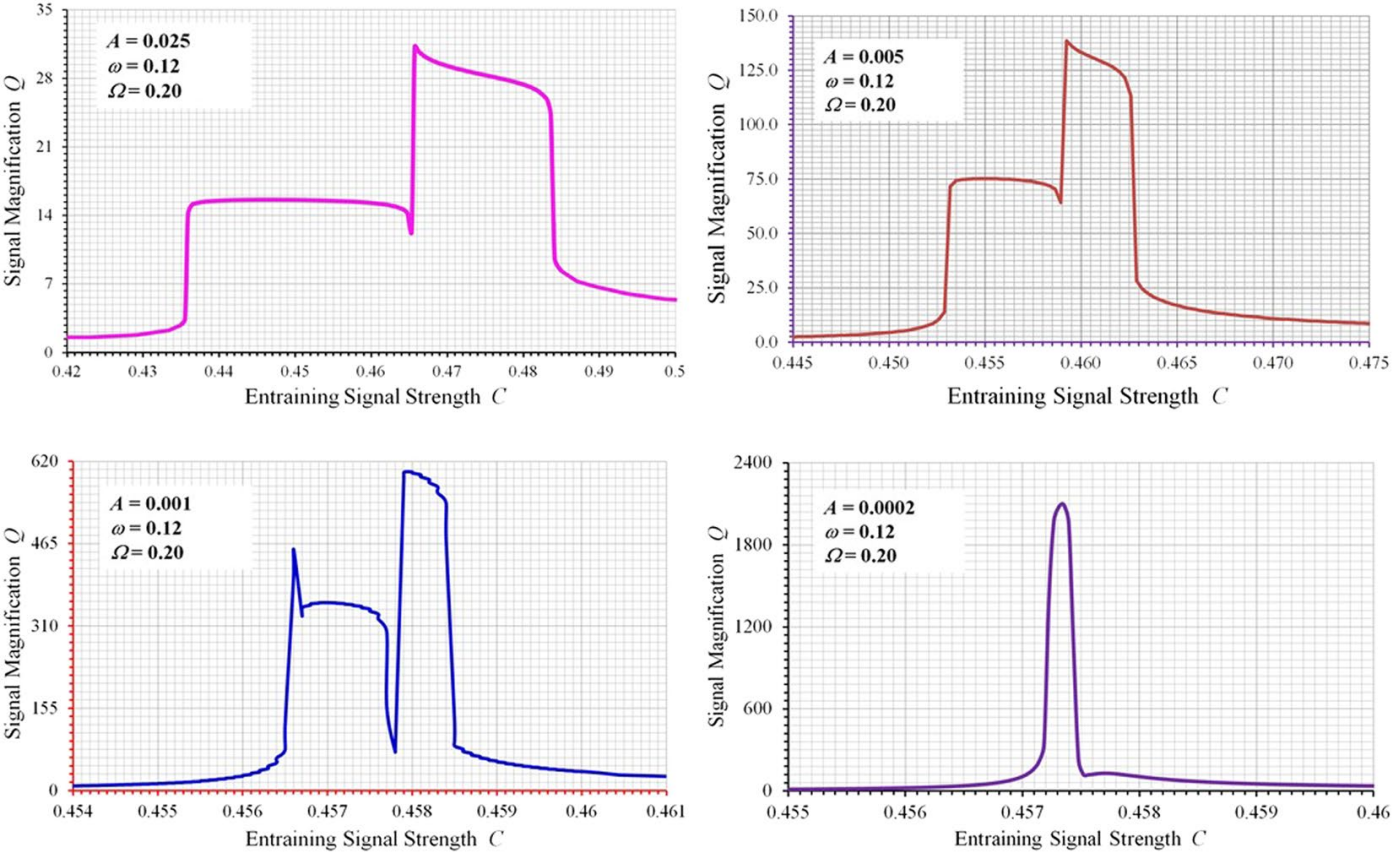
Magnification factors computed with different magnitudes of weak input signals, showing self-scaling characteristics of strong spectral resonances. (**a**) *A* = 0.025. (**b**) *A* = 0.005. (**c**) *A* = 0.001. (**d**) *A* = 0.0002.

Another important observation from our studies is the self-scaling effect of a spectral resonance. As the weak signal amplitude becomes smaller, a strong spectral resonance has the innate ability to increase the signal magnification factor *Q* in order to maintain a decent level of sensibility. This is perhaps the key mechanism behind the exquisite sensitivity of some animals to extremely weak coherent signals. The presence of the weak signal is more important than its actual strength. Once a weak input signal is there and is felt by the system, in the case of strong spectral resonance, the system will manage somehow to grow the weak signal through nonlinear modulations and intermodulations to a level that it can be adequately sensed and identified. Such a salient feature is illustrated by examining the signal magnification factors of spectral resonances under different input strengths of a weak signal. As shown in Fig. 5, the signal magnification factor *Q* increases substantially as the strength of the weak signal decreases. In the meantime nevertheless, the parameter region in which strong spectral resonance occurs becomes narrower as *Q* increases, indicating perhaps an increased difficulty in tuning the resonator to its strong spectral resonance.

### Prediction of optimal parameters of entraining signal

**Figure 6:**
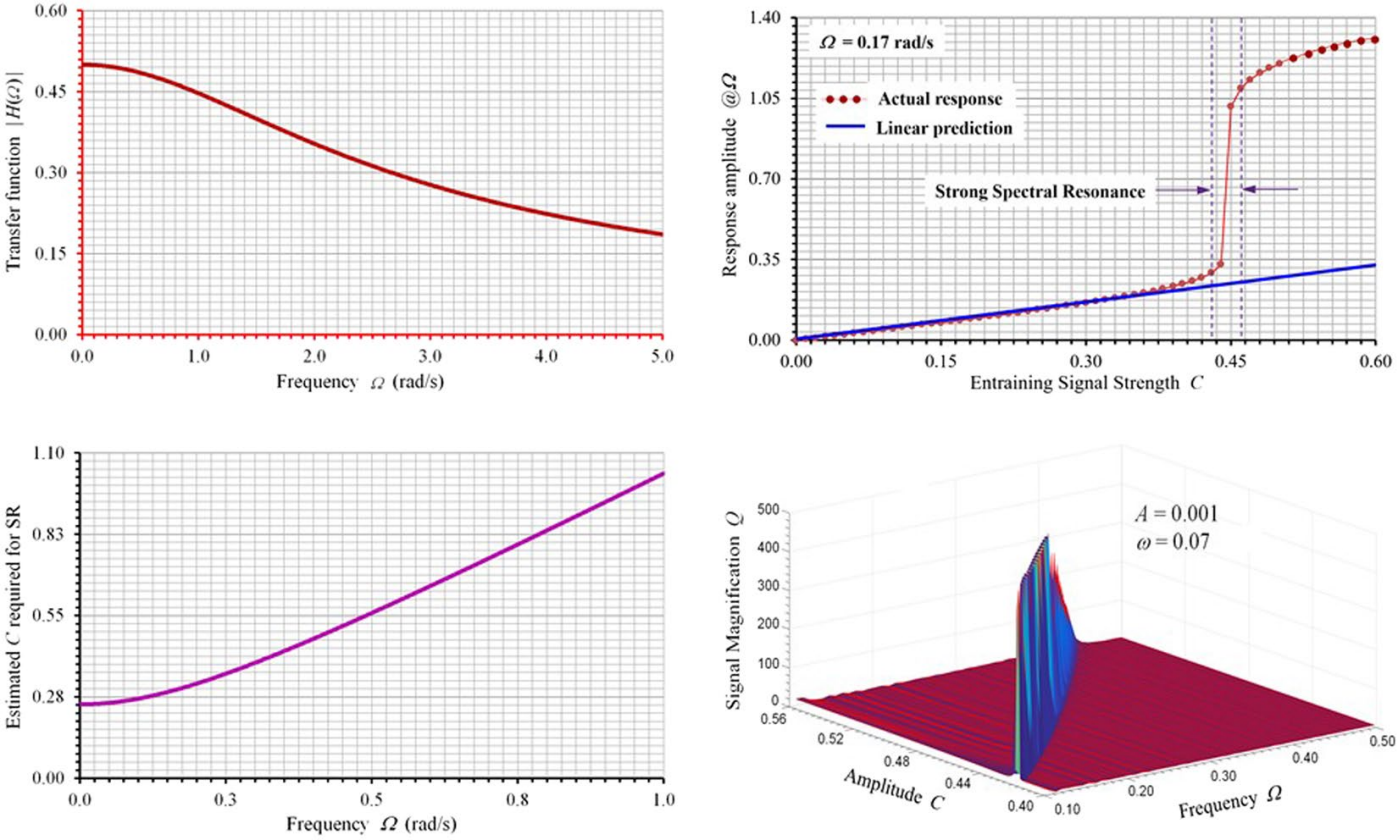
Linearized characteristics of spectral resonator and the prediction of optimal entraining parameters. (**a**) Transfer function of the linearized bistable system. (**b**) Comparison of linearly predicted and actual responses showing strong spectral resonance region. **c** Predicted optimal entraining parameters. (**d**) Numerically computed entraining parameters at specific weak signal input of *A* = 0.001 and *ω* = 0.07.

As we have shown that for every weak signal, there exists an optimal entraining signal whose amplitude *C* and frequency *Ω* can be so tuned that strong spectral resonance can be excited, leading to significant improvement of the weak signal before it can be identified. The selection of the entraining frequency *Ω* is not so critical since it is only required to be chosen in the range as that of the weak signal. However, the optimal amplitude of the entraining signal *C* needs to be determined in order to achieve best possible signal enhancement. Physically, such an optimal amplitude *C* should be so selected that it just enables the system to move out of its potential wells and, to a first order approximation, can be derived based on the theory of linearized nonlinear systems. For lower values of *C*, the system will vibrate at one of its stable equilibriums with small amplitude of vibration. The system in this case is said to be linearized and behaves effectively as a linear system with its transfer function as,

2 $$H(\Omega )=\frac{1}{{\bf{i}}\Omega +2a}$$

which is shown in Fig. 6a for the standard case of interest *a* = 1, where **i** is the complex notation ${\bf{i}}\equiv \sqrt{-1}$. Vibration response amplitude predicted based on (2) becomes very accurate for lower values of *C* until just before the onset of strong spectral resonance region as shown in Fig. 6b for a particular entraining frequency Ω = 0.17. However, substantial deviations occur around the strong spectral resonance region where the required optimal value of *C* lies. As a result, the linearized transfer function of (2) cannot be used to accurately derive the optimal value of *C*. Instead, we can use the harmonic balance method^57^ to accomplish such a task. Under the sinusoidal excitation of the entraining input, when the system just moves out of its potential wells and is oscillating between the two competing stable equilibriums, one can assume, to a first-order approximation, that the vibration response of the system be written as $x(t)=\sqrt{a/b}\,\sin (\Omega t+\varphi )$. Upon substituting this assumed solution into (1) and by equating the amplitudes of the fundamental frequency component at both sides of the equation, the following can be established,

3 $$C=\sqrt{\frac{a}{b}{\Omega }^{2}+\frac{a}{b}{(\frac{3a}{4}-1)}^{2}}$$

which is shown in Fig. 6c for the standard system of *a* = 1, *b* = 1. The predicted optimal *C* value based on (3) is only approximate due to the fact that only the fundamental frequency is considered and balanced in harmonic balance method^54^, but it becomes handy in practice to have a close value to start with to tune the resonator and it does correlate well with our numerical “experimental” results of the optimal parameter region in which true stochastic resonance occurs for the case of a weak signal input of *A* = 0.001, *ω* = 0.07, as shown in Fig. 6d.

### Method of identification of weak signals

**Figure 7:**
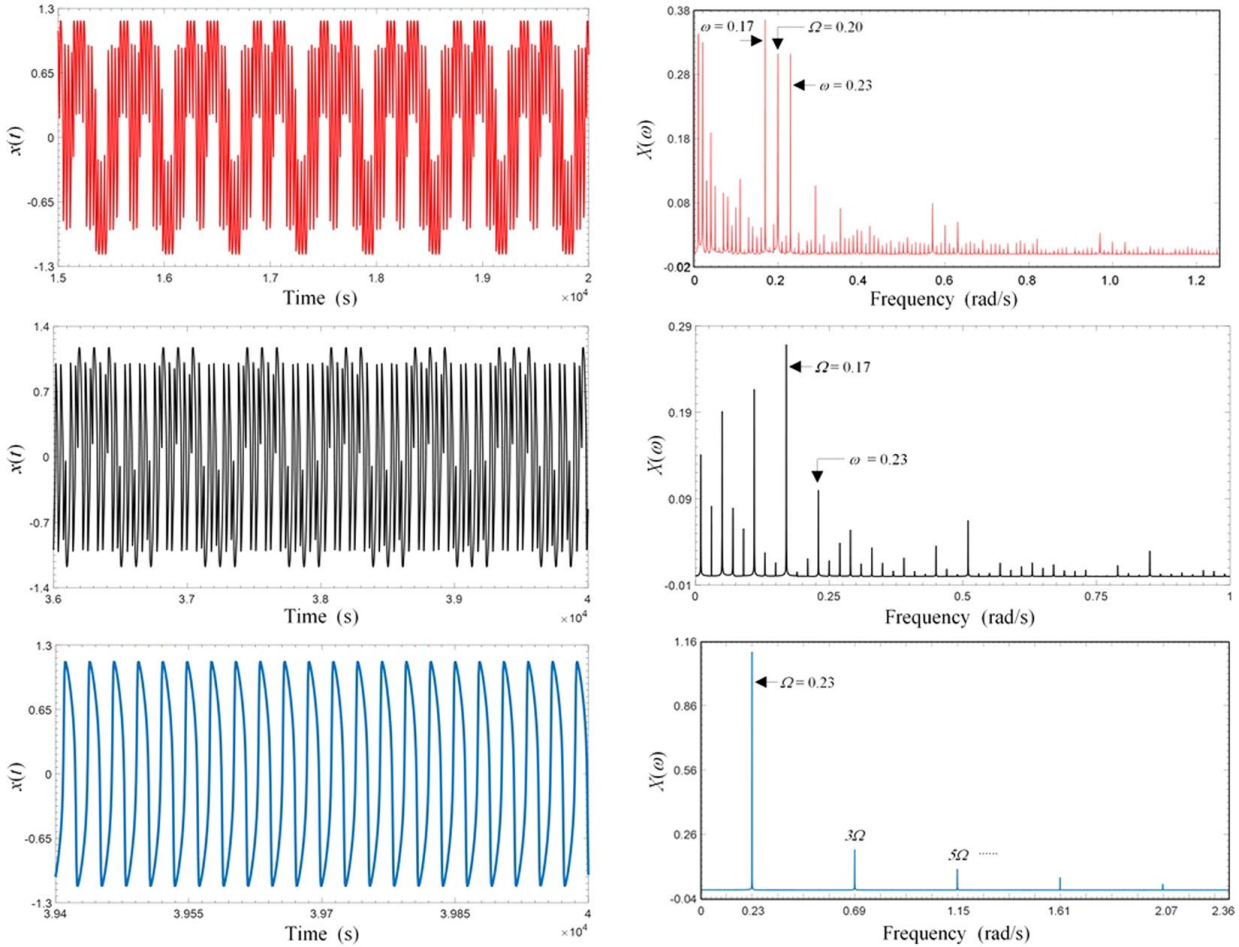
Identification of signal frequency. (**a**) Time response of spectral resonance. (**b**) Spectrum of time response in (**a**) showing prominent frequencies as possible signal frequency. (**c**) Time response of spectral resonance when entraining signal changed to one of the suspected but incorrect frequencies. (**d**) Spectrum of time response in (**c**). (**e**) Time response of a periodic signal when entraining signal changed to one of the suspected but correct frequencies. (**f**) Spectrum of time response in (**e**).

The ultimate objective is obviously to identify its frequency and magnitude once the presence of the weak signal is detected through spectral resonance. Here we propose a strategy to accomplish such a task. For every unknown weak signal to be identified, an entraining signal with a frequency in the operating frequency range is first selected. The required amplitude *C* of the entraining signal is then obtained based Eq. (3), which takes the system to the region of strong spectral resonance. Fine tune further the *C* value so that strong spectral resonance is established. Such an application is best performed with a real-time data acquisition and analysis system. One of the salient features of strong spectral resonance, as discussed earlier, is the emergence of strong sub-harmonic components present in the response spectrum, as typically shown in Fig. 4f. Under strong spectral resonance, one of the prominent spectral lines present in the overall response spectrum must be due to the weak signal, in addition to the one of the entraining frequency. To distinguish whether a selected spectral line is that of the weak signal, we can drive the system with an entraining signal at that selected frequency and if we only observe regular response spectra (no spectral resonance) by varying the amplitude, then that selected frequency must be that of the weak signal. Otherwise, we need to examine further other remaining prominent spectral lines until the desired frequency is found. Such an elimination procedure works in practice because when the two frequencies of the weak and the entraining signal become very close, the nonlinear intermodulation effect is lost, leading to responses of clean and regular spectra. Once the frequency has been identified, the actual amplitude of the weak signal can be recovered using the multi-dimensional spectral resonance characteristic transfer function ${\mathcal H} (\omega ,A,\Omega ,C)$ of the system, which is defined as the ratio between the output response and the input weak signal at the signal frequency *ω*. Such an identification process is similar to that of a linear system where measured responses are used together with system transfer functions H(*ω*) to compute the unknown input signals. However, the characterization of ${\mathcal H} (\omega ,A,\Omega ,C)$ requires more detailed measurements or numerical experiments due to its multi-dimensionality. Figure 6d shows a typical such transfer function obtained with specific weak signal frequency *ω* and amplitude *A*. To see how the proposed strategy works, consider the identification of a weak signal of *ω* = 0.23 and *A* = 0.001. First, an entraining frequency in the range is selected as Ω = 0.20. The required amplitude *C* is computed based on (3) and is then fine-tuned until strong spectral resonance occurs, as shown in Fig. 7a,b. Then the two prominent frequencies in the response spectrum (*ω* = 0.17 and *ω* = 0.23 as shown in Fig. 7b) are selected as possible signal frequency for further examinations. For the case of *ω* = 0.17, when the entraining signal input is changed to Ω = 0.17 strong spectral resonance again occurs as shown in Fig. 7c,d, ruling out the possibility that the frequency of the weak signal is *ω* = 0.17. For the case of *ω* = 0.23, when the entraining signal input is changed to Ω = 0.23, no obvious stochastic resonance occurs and the response waveform becomes regular, as shown in Fig. 7e,f, indicating that the frequency of the weak signal is indeed *ω* = 0.23. Once the frequency is confirmed, actual amplitude can be determined using the known transfer function priorly determined as discussed.

**Figure 8:**
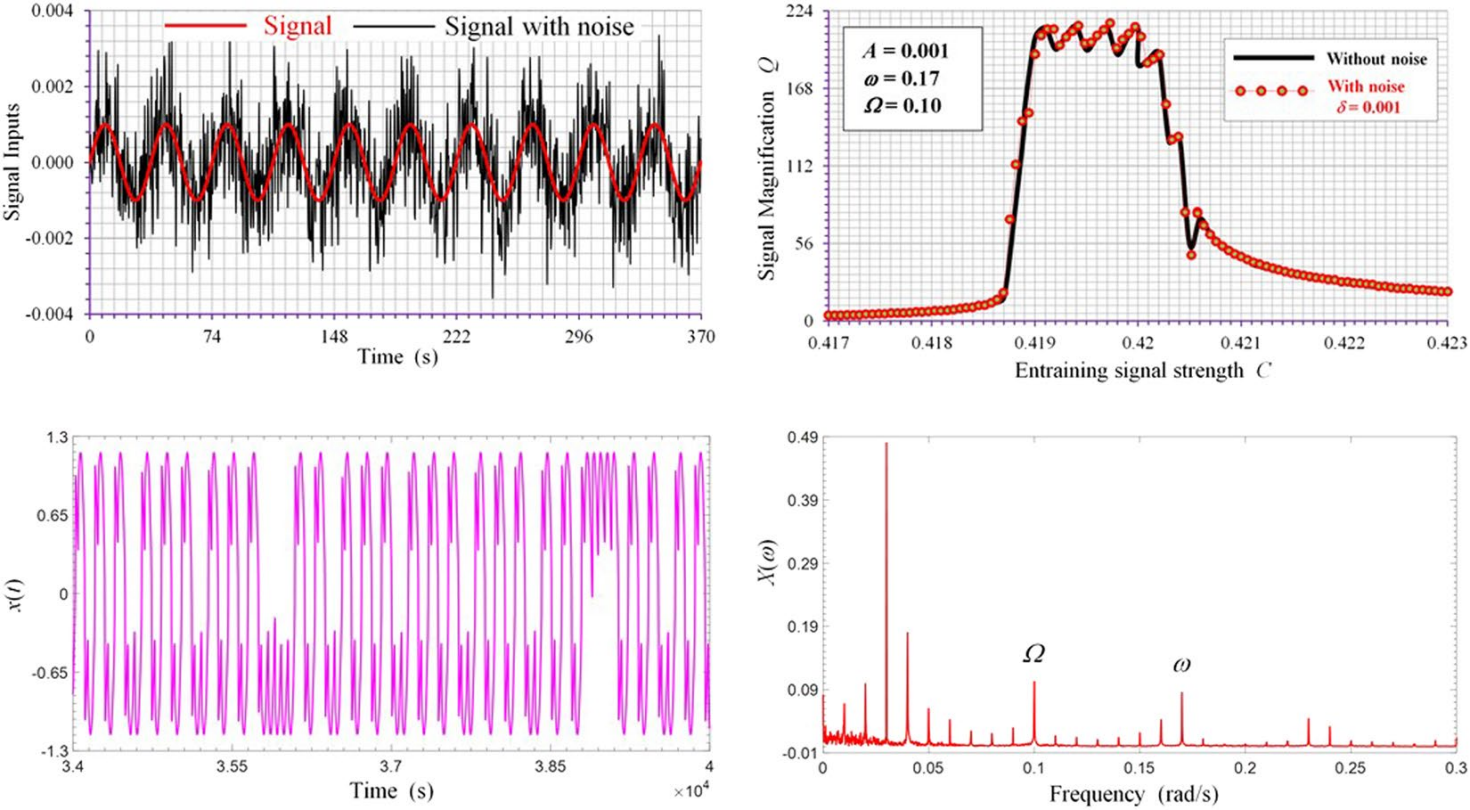
Effect of noise on spectral resonance. (**a**) Weak input signal contaminated by substantial random noise. (**b**) Comparison of spectral resonance strengths with and without noise. (**c**) Time response of spectral resonance with input contaminated by noise showing increased irregularity in wave form. (**d**) Spectrum of time response in (**c**) showing low frequency noise spectrum.

Furthermore, we examine the effect of random noise on the performance of spectral resonance and weak signal identification. The weak signal input is assumed to be contaminated by similar magnitude of Gaussian white noise so that the system equation of motion can be written as,

4 $$\dot{x}=-\,\frac{d}{dx}U(x)+A\,\sin (\omega t+\alpha )+C\,\sin \,\Omega t+\delta \xi (t)$$

where *δ* is a parameter used to control the noise level. Generally, we have observed that noise contamination on input signal does not alter significantly the spectral resonance characteristics of the system, as shown in Fig. 8b in which similar optimal signal magnification *Q* is achieved when the input signal is tainted by a considerable amount of Gaussian white noise, as shown in Fig. 8a. Nevertheless, the system response in the presence of noise becomes more irregular in waveform as shown in Fig. 8c and there appears some noticeable noise power spectrum in the low frequency range as shown in Fig. 8d, where input parameters for this case are set as *A* = 0.001, *δ* = 0.001 and *C* = 0.4195.

**Figure 9:**
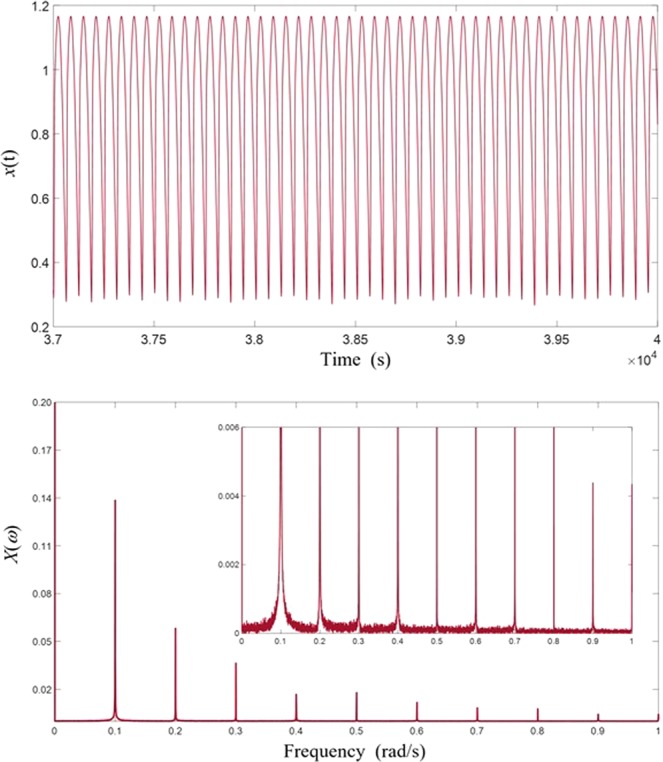
Vibration response and spectrum with random noise but no weak signal input. (**a**) Vibration response. (**b**) Response spectrum showing all harmonic components and noise contamination.

Finally, we can demonstrate that noise input signal alone, in the absence of weak signal, will not lead to misidentification. First, it is worth mentioning that the handling of random noise in numerical integration is not straightforward^58^ and some kind of low-pass filtering is always required to “smooth” the noise signal. In our numerical implementation, for a given required integration time step and time duration, a series of Gaussian random noise data points are first generated at each time step. Then, during the integration process, the values of the noise signal within each time step required during the numerical integration process are then computed using linear interpolation based on the two end-values of the time step. Such a numerical interpolation is somewhat equivalent to passing the true white Gaussian noise through a low-pass filter with a Nyquist frequency being the sampling frequency^59^. For the same input parameter setting of Fig. 8c,d but with weak input signal being removed (*A* = 0, *δ* = 0.001, *C* = 0.4195), the vibration response and its spectrum are shown in Fig. 9. We can see that the vibration response in this case becomes very regular, involving only harmonics which are generally expected. Nevertheless, since the system is being driven to the vicinity of double-well oscillation, the absence of the weak signal input has led to significant changes in the response (as compared with that of Fig. 8c), resulting in single-well oscillations and a spectrum possessing all harmonic components including DC which is obviously absent in Fig. 8d due to its largely symmetric double-well oscillation. Though the spectrum is contaminated by noise, it is clearly defined and does not indicate that there is another input signal frequency. In addition, it is interesting to note that the input noise only affects the troughs of the response curve, the peak values are not much affected, as shown in Fig. 9a.

It is worth pointing out that the frequency values of the signals investigated are low since for the particular system parameter settings of *a* = 1 and *b* = 1, the system behaves somehow like a low-pass filter and as a result, it becomes difficult to excite the system into strong spectral resonances at high frequencies. In practice, for a given range of application frequencies, the non-dimensionalized system parameters *a*, *b* and *γ* need to be accordingly tailored to facilitate better system responses, where *γ* is the damping coefficient of the general system equation of motion,

5 $$\gamma \dot{x}=-\,ax+b{x}^{3}+A\,\sin (\omega t+\alpha )+C\,\sin \,\Omega t$$

### Energy transfer between frequencies of nonlinear systems

**Figure 10:**
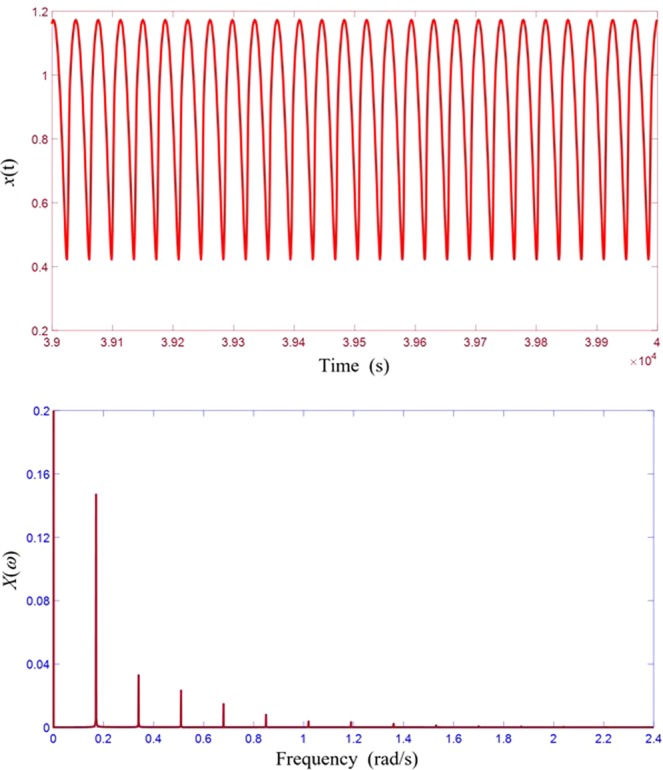
Asymmetric response showing all harmonics and DC components. (**a**) Vibration response. (**b**) Response spectrum.

We have demonstrated the existence of a new type of nonlinear spectral resonances and how it can be employed to better enable detection and identification of weak signals. It remains to explain the possible physical mechanism behind such spectral resonances. Rigorous treatment of a topic as such seems to be difficult and the authors only seek, with the help of numerical results, to provide some premature explanations in order to start some further and more fruitful discussions on this important issue. For a linear system, we all know that it possesses unique (first-order) transfer functions H(*ω*) between the system output and input which completely describe the system characteristics. In this case, a monotone (pure sinusoid) input results in a monotone output with the same frequency except that its magnitude and phase are properly determined by the transfer functions H(*ω*) at the said frequency *ω*. Hence for linear systems, there does not exist energy transfer between different frequencies. For a nonlinear system however, not only its first-order transfer functions, which represent the behavior of the linear/linearized part of a nonlinear system, but also its higher-order transfer functions, which characterize the system nonlinearities, are required in order to fully describe a nonlinear system^60,61^. The first-order transfer functions predict the vibration responses at the fundamental excitation frequencies, while the higher-order ones predict those at the harmonic and combinational frequencies^62^. A monotone input to a nonlinear system is known to lead to the spread of the input energies to harmonic frequency components. This has been shown in Fig. 4a–c for the present nonlinear bistable system. In the case of symmetric response, only odd harmonics are present (see Fig. 4a–c). However, this is obviously not always the case and for some input parameters, the vibration response can become asymmetric, resulting in all harmonics including DC components, as shown in Fig. 10a,b (Ω = 0.17, *C* = 0.44). Such observations lead us to the general modulation behavior of nonlinear systems to generate harmonic frequencies in the case of monotone inputs,

6 $${\omega }_{n}=n\Omega \,\,({n}\in Z)$$

where *Ω* is the input frequency, *Z* is a set of integers including zero. Some harmonics may not be present due to their magnitudes being zero for the particular system and input parameters under consideration.

In the case of dual-tone inputs with signal frequency *ω* and entraining frequency *Ω*, the situation becomes much more complex, not only harmonics of *ω* and *Ω* are present in the output due to modulations, but also frequency components ±*n*_1_*ω* ± *n*_2_Ω due to the intermodulations between the two frequencies where *n*_1_ ∈ *Z* and *n*_2_ ∈ *Z* are integers. Such intermodulation process then continues until all distinct frequency components are established. Again, depending on the nature of a nonlinear system and input, some of these components may be absent. So, the process of transferring energies between the frequencies in a typical nonlinear system can be summarized below:

Initial modulations to produce harmonics components:

7 $${\overline{\Omega }}_{i}=i\omega ,\,{\overline{\Omega }}_{j}=j\Omega \,(i,j=1,2,3,\mathrm{...})$$

First stage intermodulations to generate new frequency components:

8 $${\overline{\overline{\Omega }}}_{k}=\pm {n}_{ki}{\Omega }_{i}\pm {n}_{kj}{\Omega }_{j}=\pm i{n}_{ki}\omega \pm j{n}_{kj}\Omega \,\,({n}_{ki},{n}_{kj}\in Z,k\in {Z}^{+})$$

Further intermodulations to spawn more new frequencies:

9 $${\overline{\overline{\overline{\Omega }}}}_{l}=(\pm {\overline{\overline{\Omega }}}_{m})+(\pm {\overline{\overline{\Omega }}}_{n})=(\pm {n}_{mi}{\Omega }_{i}\pm {n}_{mj}{\Omega }_{j})+(\pm {n}_{ni}{\Omega }_{i}\pm {n}_{nj}{\Omega }_{j})\,\,(l,m,n\in {Z}^{+})$$

**Figure 11:**
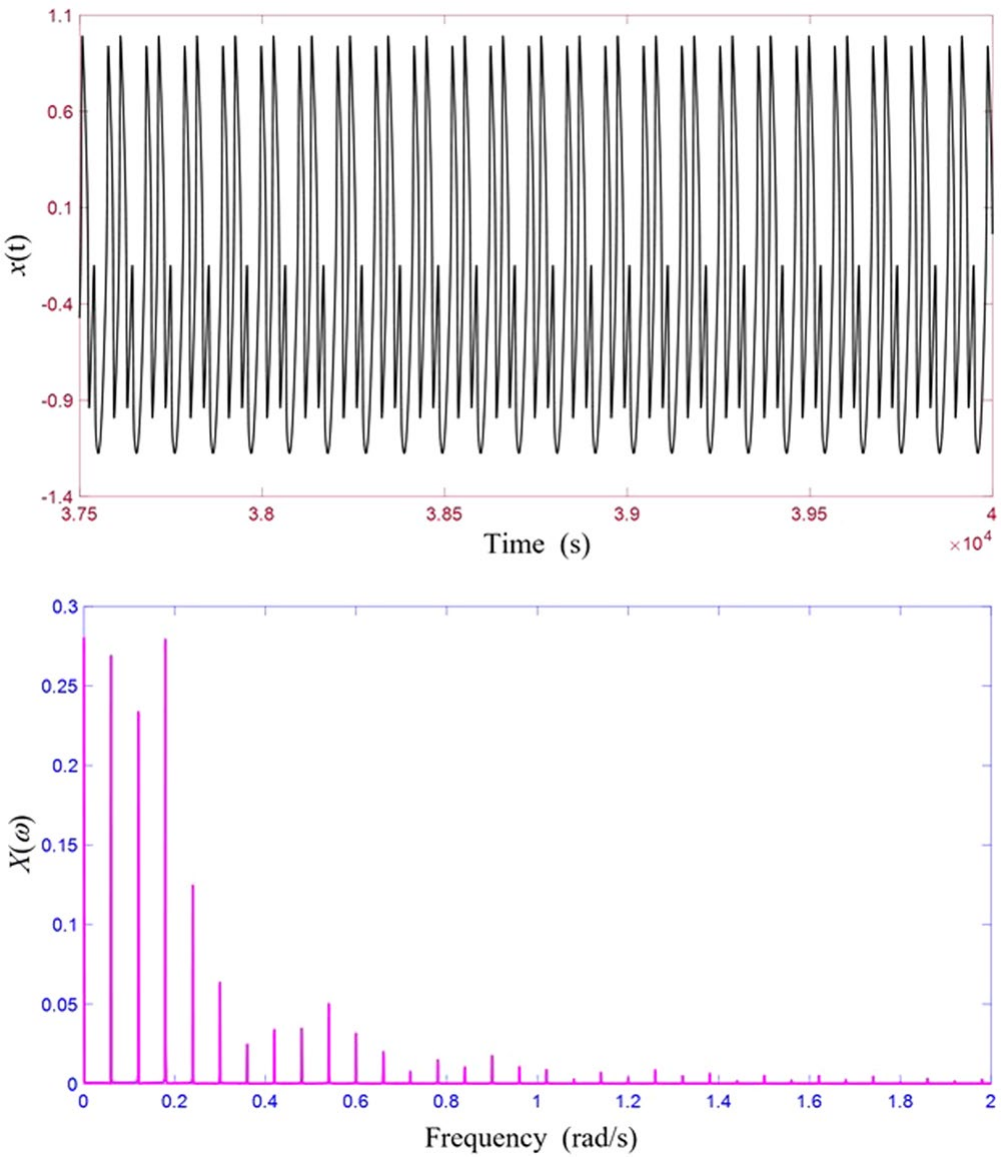
Asymmetric response and its response spectrum from dual-tone input excitation. (**a**) Vibration response. (**b**) Response spectrum.

The process continues until all distinct frequencies are produced, though some of them may be absent since their amplitudes being zero for the particular nonlinear system and forcing conditions under consideration. To briefly illustrate how this process works, we can examine the results shown in Fig. 4d–f in the case of dual-tone input. Since *ω* = 0.11 and *Ω* = 0.17, it is not possible for the modulations and intermodulations to eventually produce a DC component, since we know that 11 and 17 are co-prime numbers. The first peak is at a frequency of 0.01 which is produced through the steps: 0.17–0.11 = 0.06, 3 × 0.06 = 0.18, 0.18–0.17 = 0.01. Since there is 0.01 frequency component, we can expect all multiples of this frequency which are the peaks shown in Fig. 4f. The even multiples are absent due to the symmetric nature of the particular response. To shed more insight, we examine another case of *ω* = 0.12, *A* = 0.001, *Ω* = 0.18 and *C* = 0.45 whose vibration response and spectrum are shown in Fig. 11a,b. Since in this case the two input frequencies are no longer co-prime numbers, a DC component will be generated: 0.18–0.12 = 0.06, 3 × 0.06 = 0.18, 0.18–0.18 = 0. The smallest frequency becomes 0.06 and all the modulations and intermodulations cannot generate any frequency which is smaller than 0.06. As a result, we see a strong DC component and peaks at all the multiples of 0.06, both even and odd.

From the process of modulations and intermodulations, we can see clearly how the input energy, which is originally confined to the input frequencies is redistributed/transferred to other frequencies. Under optimal system and input parameters, many of these frequencies resulting from the modulations and intermodulations fall on the signal frequency, leading to significant enhancement of the weak signal to be extracted. On the other hand, how much energy each frequency receives depends very much on the linearized transfer functions of the system under that particular input/excitation condition^62^, since for a nonlinear system, these transfer functions are very much dependent on input (or output). As a result, by tuning properly the strength *C* of the entraining signal input, it becomes possible to obtain large desired vibration response at the frequency of the weak input signal, leading to the observed spectral resonance. Furthermore, modulations and intermodulations are better facilitated when nonlinear dynamic systems are considered, especially when detection and identification of dynamic periodic weak signals are of interest, though the existence of spectral resonances in systems with static thresholds cannot be ruled out. Further research studies are warranted in this direction. Finally, the intermodulation process requires at least two distinct frequencies to work, but questions remain whether more frequencies with more than one entraining inputs become better capable of enhancing weak signals. We are currently working on this and results will be reported in due course. Our intuitive observation is that further improvement is possible, but significant gain is less likely.

The preceding analysis can perhaps also be applied to explain the mechanism behind signal enhancement of stochastic resonances under the original setting in which the entraining signal is a white/pink Gaussian noise with strength *D*^63^. The input energy spectrum is distributive over a wide frequency range in this case and these frequency components undergo intermodulations with the frequency and its harmonics of the weak signal. The strength of the output signal at signal frequency again depends on the system transfer functions associated with the particular input, which, in turn, is a function of the input signal strength *D* of the Gaussian noise. At some optimal *D* values, signal enhancement becomes possible in which the system is said to be undergoing stochastic resonance. Physically, optimal *D* values ensure that the modulation time scale, which characterizes the dynamics of the weak periodic signal, and the escape time scale, which measures the noise induced escapes over the potential barrier, are properly matched to facilitate the growth of stochastic resonances^3^. However, due to the wide spread of the input energy, the resultant share of energy of each frequency becomes rather limited, leading to inadequate modulations and intermodulations and suboptimal signal enhancement.

## Discussions

Stochastic resonance has recently attracted massive research interests due to its enormous potentials for important practical applications. However, the current state-of-the-art of the research yet suffers very limited performance from the inappropriate choice of entraining signals. To further improve performance of existing SR, we have reported a new type of spectral resonance and how it can be harnessed for improved identification of weak signals. Such spectral resonance is much stronger than the conventional stochastic resonances discussed to date and it offers orders of magnitude better signal improvement than what is currently possible. When sinusoidal entraining signals with frequencies in the same range as that of the weak signal to be identified are employed, the underlying mechanism of spectral resonance tends to facilitate increased nonlinear modulations and intermodulations, thereby enhancing energy transfers and improving the strength and sensibility of the weak signals. For every given weak signal, there exists an optimal domain of entraining parameters to which strong spectral resonances can be tuned. We have shown that signal magnification factors in the range of thousands can be possibly achieved. An essential criterion on the onset of strong spectral resonance has been defined as the emergences of substantial sub-harmonic frequency components in the overall response spectrum, based on extensive numerical experiments. Such a criterion is practically important since it provides a definitive guide for tuning a nonlinear spectral resonator, as well as the process of identification of the weak signals. Furthermore, our studies have shown that strong spectral resonances possess unique self-scaling characteristics. As the strength of the weak input signal reduces, a true stochastic resonance has the innate ability to accordingly increase the output signal strength in order to maintain a decent level of sensibility. Such characteristics are believed to be the possible mechanism behind the exquisite sensitivity of some animals to extremely weak coherent signals.

The emergence of strong spectral resonance requires an entraining signal which has the right strength to just overcome the potential barrier and to drive the system out of its potential wells so that the additive weak signal is enabled to assist the crossings between the two competing stable equilibriums. As a result, there usually exists a narrow optimal parameter region in which a resonator can be tuned. We have developed a practically useful method which can be used to estimate the required optimal parameters. These estimated parameters become very useful and can make the tuning of a strong spectral resonance a much easier task to accomplish in practice. Though there also exists similar time-scale matching condition^2^ from which optimal noise strength *D* required for stochastic resonance in the case of entraining noise can be estimated, practical implementations become much more difficult due the randomness of the signals and above all, the performances remain much to be desired.

An effective strategy for the eventual identifications of weak signals through spectral resonances has been proposed and demonstrated. The proposed method works the best in practice with real-time data acquisition and signal processing capabilities. The system is first tuned to its strong spectral resonance to enable the weak signal to be enhanced. Signal frequency is then identified and its magnitude determined from the system response using the correlation technique. Finally, the true strength of the weak input is recovered. The process of the final recovery of signal strength can be considered as a generalization of input identifications using measured output signals and known system transfer functions which, in the present case, are multi-dimensional spectral resonance characteristic transfer functions obtained through detailed prior characterization. The effect of noise on spectral resonance has been assessed. The presence of noise leads to increased irregularity in response waveform under a strong spectral resonance and contributes noticeably to the low frequency response spectrum, but it does not substantially undermine the maximum capacity of the spectral resonator.

In conclusion, the present work seeks to stimulate some fruitful discussions on the underlying mechanisms of stochastic resonances and how to further improve their performances for better identification of weak signals. A new type of spectral resonance, which has been proven to be more capable than the existing SR, has been discussed, together with some preliminary explorations on its possible mechanism. The results have revealed the hidden potentials of spectral resonances and provided a new benchmark for future research efforts and directions. Though the analyses have been made based on bistable nonlinear systems, the fundamental mechanism and most of the established salient features are believed to be generally applicable to many other nonlinear systems known to exhibit spectral resonance behavior.

## Methods

### Numerical simulations

Numerical simulations were performed using Matlab as a computational platform. A highly accurate 5^th^-order Runge–Kutta integration scheme proposed by Dormand and Prince^64^ with tight accuracy and step size control is used for all nonlinear time-domain response predictions under various system input parameters. Matlab built-in fast Fourier transform routines are used for the computations of response spectra in frequency-domain. The extraction of a frequency component from a given response *x*(*t*) can be accomplished by using the correlation technique^60^. Suppose that a general response *x*(*t*) of a nonlinear system, which embodies a number of discrete frequency components, can be written as,

10 $$x(t)=\mathop{\sum }\limits_{n=1}^{N}{a}_{n}\,\sin ({\omega }_{n}t+{\varphi }_{n})$$

If we would like to extract the *ω*_*n*_ frequency component embedded in *x*(*t*), we can first multiply *x*(*t*) by a sinusoid at *ω*_*n*_ and numerically integrate the product,

11 $$X({\omega }_{n})=\frac{2}{T}{\int }_{\tau }^{T+\tau }x(t)[\,\sin ({\omega }_{n}t)+{\bf{i}}\,\cos ({\omega }_{n}t)]dt$$

where *τ* is a time delay to allow steady-state response to be established. Then, the modulus and phase of *X*(*ω*_*n*_) are the amplitude *a*_*n*_ = |*X*(*ω*_*n*_)| and the phase *φ*_*n*_ = ∠*X*(*ω*_*n*_) of the *ω*_*n*_ frequency component respectively. The integration of (11) is numerically carried out through summations over all the discrete time steps. Such a correlation technique has been proven to be very accurate when integration over adequate time period *T* is performed^60^.

### Derivation of linearized transfer function H(*Ω*)

When the amplitude of the entraining input signal is low, the nonlinear system vibrates with low vibration amplitude around one of its stable equilibriums and the system behaves very much like a linear system, possessing a linearized transfer function which effectively describes its dynamics. The equation of motion of (1) in this case can be written as,

12 $$\dot{x}-ax+b{x}^{3}=C\,\sin \,\Omega t$$

Since the two stable equilibriums are known to be ${x}_{m}=\pm \sqrt{a/b}$, one can assume that the overall response of the system be *x* = *x*_*m*_ + *z* where *z* represents the vibration of small amplitude around equilibrium. Upon substituting the assumed solution into (12), we have,

13 $$\dot{z}-a({x}_{m}+z)+b{({x}_{m}+z)}^{3}=C\,\sin \,\Omega t$$

14 $$\dot{z}-a{x}_{m}-az+b{x}_{m}^{3}+3b{x}_{m}^{2}z+3b{x}_{m}{z}^{2}+{z}^{3}=C\,\sin \,\Omega t$$

15 $$\dot{z}+2az+3\sqrt{ab}{z}^{2}+{z}^{3}=C\,\sin \,\Omega t$$

when *z* is small, the system becomes effectively linear after ignoring the higher order nonlinear terms as,

16 $$\dot{z}+2az=C\,\sin \,\Omega t$$

From the theory of linear systems^62^, the transfer function of the underlying linear system under small amplitude of vibration becomes,

17 $$H(\Omega )=\frac{1}{{\bf{i}}\Omega +2a}$$

which is the same as Eq. (2) referred in the main text. The linear system theory only predicts vibration response at the input frequency and for the particular system parameters studied in this paper (*a* = 1, *b* = 1), the linear transfer function *H*(Ω) = 1/(**i**Ω + 2) has a maximum amplitude of 0.5 when Ω = 0, any magnification of the input signal must come therefore from the nonlinear characteristics of the system.

### Derivation of optimal entraining signal strength *C*

For strong spectral resonance to occur, we need to first overcome the potential barrier to move the system just out of its potential wells. While reaching that strong spectral resonance region, the system will be jumping between the two stable equilibriums and hence its vibration response, to a first order approximation, can be written as $x(t)=\sqrt{a/b}\,\sin (\Omega t+\dot{\varphi })$. Upon substitution of this assumed solution into (12), we have,

18 $$\sqrt{a/b}\Omega \,\cos (\Omega t+\varphi )-a\sqrt{a/b}\,\sin (\Omega t+\varphi )+b{(\sqrt{a/b})}^{3}{\sin }^{3}(\Omega t+\varphi )=C\,\sin \,\Omega t$$

Using the trigonometric identity,

19 $${\sin }^{3}(\Omega t+\varphi )=\frac{3}{4}\,\sin (\Omega t+\varphi )-\frac{1}{4}\,\sin (3\Omega t+3\varphi )$$

the following can then be obtained,

20 $$\begin{array}{c}\sqrt{a/b}\Omega \,\cos (\Omega t+\varphi )-\sqrt{a/b}\,\sin (\Omega t+\varphi )+\frac{3a\sqrt{a/b}}{4}\,\sin (\Omega t+\varphi )\\ \,\,\,\,\,\,\,\,\,\,\,\,\,\,\,\,-\frac{a\sqrt{a/b}}{4}\,\sin (3\Omega t+3\varphi )=C\,\sin \,\Omega t\end{array}$$

Based on harmonic balance method^65^ which requires the fundamental frequency *Ω* components on both sides of (20) be equal, we can then obtain the required strength *C* of the entrained signal as,

21 $$C=\sqrt{\frac{a}{b}{\Omega }^{2}+\frac{a}{b}{(\frac{3a}{4}-1)}^{2}}$$

which is the same as Eq. (3) referred in the main text.
